# Dr. Kinglake, on the Medicinal Effects of Digitalis

**Published:** 1800-02

**Authors:** Robert Kinglake

**Affiliations:** Bristol


### *To the Editors of the Medical and Phyſical Journal.*

Gentlemen,

It falls to the ſhare but of few medicinal agents to engage ſo much zeal and acuteneſs of obſervation, as have been already beſtowed on Digitalis; and but few, indeed, hitherto diſcovered, have had equal claim to attentive inveſtigation.

It could have been wiſhed, however, that more unanimity in this inquiry had leſs equivocally determined its powers and mode of agency.

Your Journal has been the channel of various opinions on this intereſting reſearch; it probably may not, then, be inconſiſtent with your deſire for diverſity of intelligence on the ſame ſubject, to add to the preceding obſervations of your correſpondents, a brief recital and compariſon of facts, which do not appear to have been yet diſtinctly noticed.

Dr. Moſſman's obſervations on the effects of digitalis in pulmonary pulmonary confumption*, feem to have been made with equal impartiality and judgment; and though the general refult of this practice has been lefs favourable than has occurred in fome other inftances (yet it is, perhaps, countenanced by the moft prevailing experience), ftill more fuccefs would have probably accrued from the treatment employed, if fmall quantities of folid animal food, at fhort intervals, had been fubftituted for the more fluid and lefs nutritious fubftances, milk and eggs. Thefe are, indeed, not objectionable; but ample experience has fully convinced me, that portions of either pork, mutton, or beef, not exceeding one ounce at a time, rather under dreffed, well mafticated, and repeated about every two hours until fix o'clock in the evening, then difcontinuing the courfe, and refuming it at about eight the following morning, will prove, in every ftage of pulmonary confumption, abundanty more ftrengthening and falutary.

On the powder having repeatedly difappointed expectation, the reputation of the tincture, the decoction and infufion (more particularly that of the firft) fhould have refpectively entitled them to a comparative trial. With me, the powder has, in moft inftances, either fpeedily naufeated, or inordinately increafed the renal and inteftinal evacuations; and, under fuch circumftances, has wholly failed to amend the pulmonic diforder.

The experience of Dr. Bree† has been fingularly adverfe to the falutary efficiency of Digitalis in phthifis pulmonalis. As no effect can happen, however, without a caufe, fo the ground of this almoft uniform failure refts moft properly with the Doctor himfelf to explain. Induction from extenfive analogy refufes to admit, that the digitalis was either abfolutely inefficacious, or neceffarily noxious. In upwards of one hundred cafes, in which I have lately had occafion to prefcribe it, it has nearly as invariably alleviated the prevailing fymptoms, and in no inftance did it appear to be deleterious. Two in three, at the loweft computation, in every ftage of pulmonary confumption, have obtained temporary benefit from it; one in three in the firft and fecond, or in the catarrhal and tubercular ftages, has been cured; and even in the laft period of this difeafe, the exifting fymptoms have been greatly ameliorated. Much promifing amendment of feveral weeks duration has, indeed, been produced, but in no inftance has an ultimate cure been effected.

The

* Vide Medical and Phyfical Journal, Vol. II. p. 33, 238.

† Vide Medical and Phyfical Journal, Vol. II. p. 314, 430.

The precautions ſuggeſted by Dr. Sherwen,* relative to the uſe of digitalis, are more honourable to his humanity than demonſtrative of the correctneſs of his experience. To adopt them would be to ſhackle its due adminiſtration with hurtful timidity.

The ſaturated tincture of digitalis has been taken, under my direction, by children of eight years of age, in doſes of five drops, gradually increaſed to fifteen, three times a day; and by adults, from ſixty to ſeventy years old, from ten drops, ſlowly augmented to thirty, at equally ſhort intervals, during three or four weeks ſucceſſively, without the leaſt detriment, or ſlighteſt cauſe for alarm.

If the practical rule be duly obſerved, of beginning with five drops of the tincture, one-quarter of a grain of the powder, one tea-ſpoonful of either the decoction or infuſion, for children; and ten drops of the tincture, one grain of the powder, and one table ſpoonful of either the decoction or infuſion, for adults, in both caſes repeated three times a day, at equal diſtances, gradually extending the doſe, if no diſagreement enſue, or as the exigency of curative efficiency may require, diminiſhing or wholly ſuſpending the medicine if either nauſea, vertigo, or irregularity of the pulſe ſupervene; the weakeſt conſtitution, and moſt morbidly irritable temperament, may be, in general, ſafely and adequately charged with its influence.

I am perſuaded the beneficial effects of digitalis are often prevented, and the worſt inconveniences of its operation incurred, by either not commencing its employ in ſufficiently ſmall doſes, or in advancing them too rapidly. If its agency be abruptly or violently obtruded on the ſtomach, it will induce indirect debility on that organ, accompanied with a revolting nauſea to its ſubſequent adminiſtration; the effects of which will be ſpeedily propagated over the ſyſtem, and predominantly felt on the lungs.

It has occurred to me more than once, to witneſs and regret theſe pernicious conſequences from its undue exhibition.

Dr. Maclean deſerves well of ſcience and mankind, for his many excellent practical remarks on Digitalis. His communication on that ſubject† diſplays ſuperior truth, perſpicuity, and intelligence; but this juſt tribute of approbation does not diſarm my oppoſition to what experience has convinced me is exceptionable in his opinions.

Reiterated obſervation fully authoriſes me to diſſent from Dr. Maclean

* Vide Medical and Phyſical Journal, Vol. II. p. 175.

† Vide Medical and Phyſical Journal, Vol. II. p. 113.

Maclean, in his remark that naufea rather promotes than impedes the falutary agency of digitalis.

The reverfe has uniformly appeared to me to be the fact; by its increafing the prevailing general debility of the fyftem, the pulfe has been rendered fmall, weak, and hard; the cough more urgent, with lefs thoracic ability to endure its attendant exertion; harraffing laffitude, more frequent and durable febrile exacerbations, and nocturnal fweats, have alfo obvioufly rifen on the ruins of ftrength, induced by the fickening influence of digitalis. The cafes of the firft and fecond ftages of phthifis pulmonalis under my direction, in which it has failed to render permanent fervice, have been almoft without exception, thofe in which ficknefs became an early effect of its operation.

After this injurious occurrence, in vain have I in general fought to obtain that firm, full, foft, and equal pulfe, which is a much lefs fallible harbinger of radical benefit from digitalis, than its mere retardation. With reduced frequency of pulfe, (when morbidly exceeding the natural ftandard) are neceffarily affociated proportionate fullnefs and foftnefs, conditions lefs feparable from the beneficial agency of digitalis than increafed flownefs.

I have had frequent occafion to obferve much amendment afforded by it, without fenfibly retarding the pulfe; and in one tuberculous cafe, a fpeedy and perfect cure was effected without perceptibly diminifhing its celerity; it continued invariably at ninety ftrokes in a minute; the only change induced, was from hard, weak, and fmall, to foft, firm, and full; a difference effential to permanent recovery, in all ftages and circumftances of pulmonary confumption.

It will be found on trial, that the affiduous employ of nutritious diet, conjoined with frequent and brifk friction over the region of the ftomach, performed with a flefh brufh, will greatly fortify the fyftem, and contribute to obviate the diftreffing naufea, (and its difaftrous confequences) incident to the agency of digitalis. Were I to be afked what concomitant rule in the ufe of digitalis, appeared to me the moft important, my prompt reply would be, to nourifh with fmall quantities of aliment, confifting chiefly of animal food, repeated at fhort diftances, fo as not to induce either oppreffion or indigeftion, and to fupport the general ftrength, without either molefting or exhaufting it by inordinate or defultory excitement, with a view at once to refift a concentration of the influence of digitalis on the ftomach, and to fecond its falutarily ftimulant operation on the fyftem.

My opinion of the modus operandi of digitalis, is founded on a principle of lymphatic circulation, that wholly rejects the prevailing but gratuitous notion of the lymphatic veſſels abſorbing and propelling, by virtue of any independently active or contractile power, attributing the tranſmiſſion of lymph entirely to the conjoint influence of arterial and muſcular impulſe; but this ſubject will be farther explained on another occaſion.

If Dr. Maclean ſhould not have hitherto been in the habit of allowing his conſumptive patients, under the uſe of digitalis, the ſalutary excitement of moderately ſtimulant nouriſhment, he may with ſome confidence, in my experience of its utility, advantageouſly incorporate it with his code of other judicious regulations.

The tincture of digitalis, prepared from the recent leaves, as propoſed by Dr. Maclean, promiſes to be more efficaciouſly impregnated with the native virtues of the plant than that made from the dry. The ſoporific effect often produced by digitalis, and the ſtriking analogy of its general operation to that of opium, hemlock, night-ſhade, hen-bane, &c. clearly evince its narcotic property, and ſuggeſt the probability, that this quality conſtitutes the chief ground of its medicinal efficiency. It is not preciſely known, on what arrangement of elementary and conſtituent principles, the narcotic power of vegetable ſubſtances depends; but it is notorious, that it reſides moſtly in the volatile parts, and that the decompoſing influence of light, and the proceſs of exſiccation, greatly derange and diſſipate thoſe active materials.

The form of tincture is alſo, indiſputably, equally efficacious with any other ſhape in which digitalis can be employed; and, in my obſervation, indeed, has proved more ſpecifically active on the lungs, than either the powder, infuſion, or decoction, which have all a greater or leſs tendency to operate more particularly on either the ſtomach, inteſtines, or kidneys.

I will attempt no other comment on the opinions and practice of Dr. Drake,* relative to digitalis, than that which a compariſon of facts irreſiſtably furniſhes. With due deference to the talents of that practitioner, and alſo to his avowedly firm perſuaſion that he has thrice ſucceeded in curing the ultimate, or ulcerated ſtage of phthiſis pulmonalis, by the agency of digitalis, I muſt aſſume the liberty of ſuſpecting that the advanced progreſs of the tubercular, or ſecond ſtage of that diſeaſe, has deceived him into an idea of its having been the laſt period; a miſtake extremely incident to the beſt powers of diſcrimination.

My ſuſpicion is far from being either captious or illiberal, it reſts on the broad baſis of his own aſſertion, that he has never exhibited

* Vide Medical and Phyſical Journal, vol. ii. p. 267. 417.

exhibited digitalis in the tubercular ftage of pulmonary confumption; that it has proved curative under his direction in the ulcerated ftate of that malady, in three inftances of five; though I have employed it in upwards of twenty apparently fimilar occafions, with the utmoft perfeverance, and occafional variation of form, without having refcued one patient, while feven tubercular cafes (three of which were accompanied with very fpecious ulcerous circumftances) have been radically cured under my care, by its falutary influence; that, probably, the collective experience of the medical practice of Great Britain, with the folitary exception of that of Dr. Fowler, cannot afford an equal extent of fuccefs; and finally, that it is impoffible to diftinguifh the nice fhades by which the eve of the fecond and the dawn of the third ftages are feparated.

The expectorated fluid, generated by the morbid fecretion obtaining on the mucous membrane inverting the bronchial cavities, and occupied in the unbroken tuberculous ftructure, may be occafionally tinged with blood, evolve fetor, and in all refpects affume an impofing refemblance to that which is afforded by the ulcerated furface. Neither does the general or hectic ftate of the fyftem (though hypothetically imagined to be characteriftically diverfified by the equally gratuitous notion of the abforption of aerated matter) prefent any other difference than that which confifts in an occafional variation in degree of violence, and on which no practical diagnofis can be juftly founded. Nor is the duration of the difeafe an adequate datum for eftimating its exifting ftage, the progrefs being neceffarily various, in diffimilar temperaments, and under different circumftances; yet it may be fafely admitted, as a lefs ambiguous criterion than that which is fupplied by either the general or particular indication of the attendant fymptoms.

If three months be allowed for the completion of each ftage, nine months will comprife the whole period in which pulmonary confumption paffes through every gradation, from its moft incipient and flight form, to its moft confirmed and inveterate advancement; and it is accordingly found, that digitalis but rarely renders more than temporary relief in this difeafe, when it is more than fix months ftanding, and that its moft curative agency is exerted in the interval from the fecond to the fourth month. It is obvious, that afthmatic and other chronic affections of the lungs, diftinguifhable by no definite mode of progrefs, or length of duration, (but in which digitalis alfo often operates very falutarily) ought not to be confounded with real phthifis pulmonalis, and oppofed to this confideration.

Since, then, it is impoffible pofitively to afcertain that pulmonary confumption is at the termination of the fecond, or actually advanced advanced into the third ſtage, how is it to be probably preſumed? Surely, neither by haſty conjecture, nor by inferences deduced from queſtionable premiſes; but rather by the analogous efficacy of ſimilar remedies acting in parallel circumſtances.

If it might be aſſumed, (what indeed inquiry would undoubtedly verify) that the aggregate experience of medical practitioners could adduce one thouſand well-authenticated caſes of ſtrongly marked tuberculous conſumption having been cured by digitalis, and (with the exception before noticed) not one of its having ſucceeded in what is ſtrictly denominated the laſt ſtage of that malady, is it being too ſceptical in refuſing aſſent to a comparatively very limited experience, which affirms the converſe of the greater evidence, alleging that it has cured three in five in the ulcerous, and has never been employed in the tuberculous ſtate of the diſeaſe?

Dr. Drake has great merit in having cured what I eſteem to be no more than the tuberculous, or ſecond ſtage of pulmonary conſumption; if, however, the ſequel of his experience ſhould prove that I have unjuſtly ſuſpected the accuracy of his diſcrimination, he ſhall receive my cordial recantation, and will be farther amply entitled to the laſting gratitude of mankind.

It has recently occurred to me, to witneſs the curative efficiency of digitalis ſignally manifeſted in two caſes, nearly ſimilarly circumſtanced, of apparent meſenteric obſtruction.

The one was a weakly conſtituted woman, about 25 years of age, in whom general debility, progreſſive emaciation, nocturnal perſpiration, tranſient pains in the abdomen and thorax, accompanied with hectic fever, had prevailed upwards of two months.

The other was a girl, aged about twelve, of a ſcrophulous habit, in whom the preceding ſymptoms, though in a leſs degree, added to thoſe of dyſpepſia and anorexia, had exiſted about ſix weeks. In both inſtances, a cure was accompliſhed under its uſe within one month.

Conjoined with a well-regulated diet, it promiſes to be eminently efficacious in atrophical affections, originating from deficient arterial and muſcular energy, inducing lymphatic obſtructions, vitiated aſſimilation, and an inadequate ſupply of alimentary principles to the ſyſtem.

Neither in chloroſis, epilepſy, nor in external ſcrofula, has, hitherto, under my obſervation, any evident amendment reſulted from its ſedulous employ.

I am, GENTLEMEN,

Your's, reſpectfully,

ROBERT KINGLAKE.

*Briſtol, December* 14, 1799.

